# Ozone impact from solar energetic particles cools the polar stratosphere

**DOI:** 10.1038/s41467-022-34666-y

**Published:** 2022-11-12

**Authors:** Monika E. Szela̧g, Daniel R. Marsh, Pekka T. Verronen, Annika Seppälä, Niilo Kalakoski

**Affiliations:** 1 Space and Earth Observation Centre, Finnish Meteorological Institute, Helsinki, Finland; 2 Climate and Global Dynamics Laboratory, National Center for Atmospheric Research, Boulder, CO USA; 3 Faculty of Engineering and Physical Sciences, University of Leeds, Leeds, UK; 4 Sodankylä Geophysical Observatory, University of Oulu, Sodankylä, Finland; 5 Department of Physics, University of Otago, Dunedin, New Zealand

**Keywords:** Atmospheric dynamics, Atmospheric chemistry

## Abstract

Understanding atmospheric impacts of solar energetic particle precipitation (EPP) remains challenging, from quantification of the response in ozone, to implications on temperature. Both are necessary to understand links between EPP and regional climate variability. Here we use a chemistry-climate model to assess the importance of EPP on late winter/spring polar stratosphere. In transient simulations, the impact on NO_*y*_, ozone, and temperature is underestimated when using EPP forcing from the current recommendation of the Coupled Model Intercomparison Project (CMIP6). The resulting temperature response is largely masked by overall dynamical variability. An idealised experiment with EPP forcing that reproduces observed levels of NO_*y*_ results in a significant reduction of ozone (up to 25%), cooling the stratosphere (up to 3 K) during late winter/spring. Our results unravel the inconsistency regarding the temperature response to EPP-driven springtime ozone decrease, and highlight the need for an improved EPP forcing in climate simulations.

## Abstract (web summary)

This work reveals a significant energetic particle precipitation (EPP) impact on ozone and stratospheric temperatures during late winter/spring and highlights the need for an improved representation of decadal EPP forcing in climate simulations.

## Introduction

In recent years, a significant amount of scientific interest has been directed towards understanding the influence of energetic particle precipitation (EPP) on regional climate variability over decadal time scales. It is now well established that EPP affects ozone in the polar regions^1–6^. Thus, it might provide an important link to atmospheric dynamics, and play a role in modulating regional scale ground-level climate. Several observational and model studies have already proposed a link between EPP and tropospheric temperature and pressure variability^7–15^. However, the details of the timing, and mechanisms linking EPP-driven chemical impacts to tropospheric variability remain under investigation. There are particular questions regarding impacts on the stratosphere, which could provide clues on signal propagation similarities with the so-called top-down mechanism that connects solar ultraviolet irradiance, stratospheric ozone, and climate^16,17^. With the now established role of the stratosphere in seasonal climate predictions^18^ understanding of these linkages are critical for true representation of solar forcing in seasonal scale changes.

EPP constantly affects the Earth’s atmosphere. There are several types of EPP, characterized by their solar and magnetospheric drivers which define EPP energy and atmospheric penetration depth. Energetic electron precipitation consists of auroral electrons, medium energy electrons (MEE), and relativistic electrons that penetrate into the lower thermosphere, mesosphere, and upper stratosphere, respectively^19^. Solar proton events (SPEs) are more sporadic than electron precipitation but consist of highly energetic protons that can precipitate down to the upper stratosphere^20^. Galactic cosmic rays (GCR), originating from outside of our solar system and modulated by solar activity, have highest energies and thus mainly affect the lower stratosphere and troposphere.

In the polar regions, auroral electrons are an important source of NO_*x*_ (N, NO, NO_2_) species in the thermosphere, while MEE and SPE produce HO_*x*_ (H, OH, HO_2_) and NO_*x*_ in the mesosphere and the upper stratosphere^2^. The GCR effect on tropospheric/lower stratospheric NO_*x*_ is less than 1%^21^. HO_*x*_ is the main catalyst of ozone loss in the mesosphere and drives most of the direct chemical impact from EPP. NO_*x*_ effectively destroys ozone in the upper stratosphere, particularly in winter when large amounts of EPP-NO_*x*_ are transported down from above inside the polar vortex^11^. At lower altitudes, EPP-NO_*x*_ is partly converted to other species of the reactive nitrogen family NO_*y*_ (NO_*x*_, HNO_3_, N_2_O_5_, ClONO_2_, HNO_4_)^22^.

The descent of EPP-NO_*y*_ driving stratospheric ozone loss during late winter/spring, known as the “EPP-indirect effect"^23^, is a potential way by which EPP chemistry impact can initiate a response in atmospheric dynamics (less ozone → less shortwave-heating → stratospheric cooling). However, a study using 40 years of reanalysis data found a positive temperature anomaly in the springtime stratosphere^24^, which is opposite to what is expected from direct radiative cooling caused by EPP-driven ozone reduction and points to a dynamical cause.

Here, we investigate this apparent inconsistency between EPP-driven ozone and temperature responses in late winter/spring. Because 40 years of reanalysis data might not be enough for detection of the temperature signal, we overcome this by analyzing an ensemble of simulations to increase the statistical robustness of our analysis. We utilize the Whole Atmosphere Community Climate Model (WACCM)^25,26^ to study the link between the EPP-NO_*y*_ and dynamical variability in the stratosphere, with a focus on the important role of an adequate description of MEE in chemistry-climate models. Our analysis highlights the variability in the EPP-NO_*y*_ descent into the stratosphere during polar winter, the resulting impact on springtime ozone levels, and the following radiative and dynamical effects. In our analysis we utilize transient simulations for the time period 1957–2005, and idealized simulations with enhanced MEE forcing to match the satellite-based observations of stratospheric NO_*y*_^22^.

## Results

### Transient simulation of 1957–2005

**Fig. 1:**
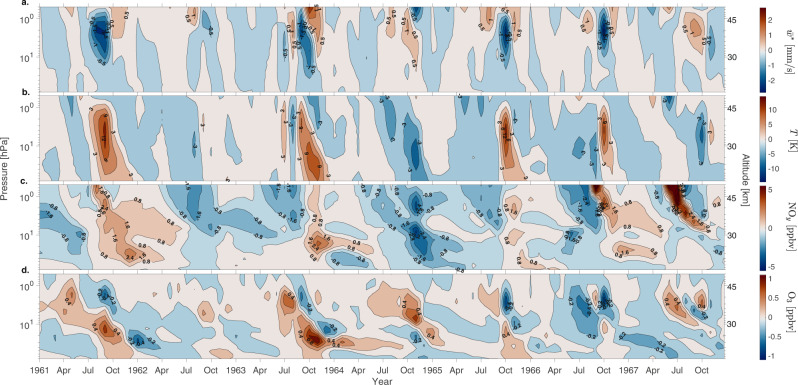
Representative annual stratospheric variability. Monthly mean anomalies in (**a**.) residual vertical wind, ${\overline{w}}^{*}$ (0.5 mm s^−1^ contour interval), (**b**.) temperature, *T* (3 K contour interval), (**c**.) nitrogen family, NO_*y*_ (NO_*x*_, HNO_3_, N_2_O_5_, ClONO_2_, HNO_4_) (0.8 ppbv contour interval), and (**d**.) ozone, O_3_ (0.2 ppmv contour interval), averaged over latitudinal range 60–90°S. Pressure levels on the *y*-axis are 0.5–80 hPa, with approximate altitude in km given on the right-hand side. Contour color scales for each panel are given on the right hand side.

Figure 1 shows an example of monthly mean anomalies in the residual vertical wind (${\overline{w}{}^{*}}$), temperature (*T*), NO_*y*_ and ozone from Southern Hemisphere and one ensemble member of the historical run for the period of 1961–1967. The years in the figure are shown as a representative example of the declining phase of the solar cycle (1961–1962), solar minimum (1963–1965), and its ascending phase (1966–1967). For all simulations, residual vertical wind, ${\overline{w}}{}^{*}$ is calculated every time step using Eq. (1) (see “Methods”). The upper stratosphere shows a clear correlation between downward (negative) ${\overline{w}}{}^{*}$ and increases in temperature as a result of dynamical heating from enhanced downwelling. This connection is clearest during late winter in 1961, 1963, 1965 and 1966 when ${\overline{w}}{}^{*}$ anomalies reach 2–2.5 mm/s (Fig. 1a) and temperature anomalies reach 12–15 K (Fig. 1b). During these winters, NO_*y*_ is efficiently transported downwards, resulting in significant increase in NO_*y*_ mixing ratio by 2.4–5.6 ppbv below 1 hPa (45 km). Where strong downwelling occurs, NO_*y*_ anomalies in excess of 1.6 ppbv extend as low as 20 km (e.g., in 1961, 1963 and 1966). Enhancements in NO_*y*_ correspond to ozone loss of about 0.2–0.8 ppmv at altitudes below 1 hPa (45 km) in October–April (Fig. 1d). The strongest negative ozone response is seen in years 1961, 1963 and 1966 when ${\overline{w}}{}^{*}$ is strongly negative and the amount of NO_*y*_ is high.

**Fig. 2:**
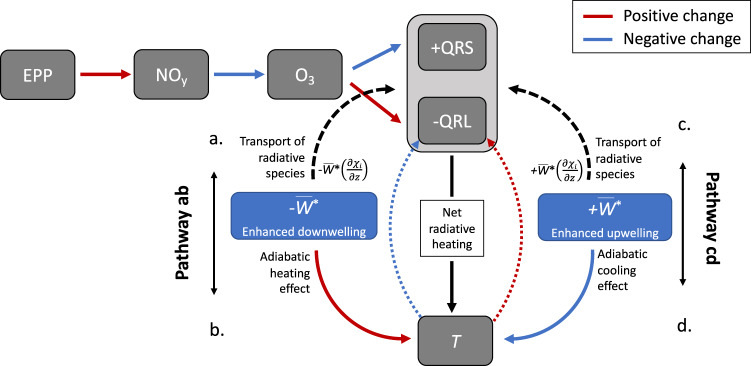
Chemical and dynamical heating contributions in the stratosphere. Schematic diagram of the potential interactions between chemical and dynamical heating contributions to temperature *T*, as a result of changes to ozone (O_3_) and up/downwelling. Red arrows indicate an increasing effect, while blue arrows indicate a reducing effect. EPP is energetic particle precipitation, NO_*y*_ is nitrogen family (NO_*x*_, HNO_3_, N_2_O_5_, ClONO_2_, HNO_4_), QRS is short-wave radiative heating, QRL is long-wave radiative cooling, ${\overline{w}}{}^{*}$ is the residual vertical wind anomaly and *χ*_*i*_ is mixing ratio of the radiatively reactive gas. Pathways ab and cd highlight the dynamical effect on temperature both directly, and via feedback to radiative cooling (marked with blue and red dotted lines).

As we can see in Fig. 1, the annual variability across all variables is large and highly influenced by dynamics. To better understand how the combined effects of EPP and dynamical variability are reflected in stratospheric temperatures several physical mechanisms must be considered (illustrated in Fig. 2). The loss of ozone following a transport-driven enhancement of EPP-NO_*y*_ will affect both short-wave heating rates (QRS) and long-wave cooling rates (QRL), since ozone both absorbs and emits radiation. Ozone loss leads to a decrease in the absorption of ultraviolet radiation that will act to cool the stratosphere. However, the effect an ozone decrease will have on QRL is the opposite above 30 km. To add to this, enhanced downwelling (Fig. 2, Pathway ab) will act to dynamically heat the stratosphere. The vertical transport that can bring down EPP-NO_*y*_ will also modify the distribution of radiatively active species. This process depends on the sign of the vertical gradient in the constituents mixing ratio (∂*χ*_*i*_/∂*z*), and so vertical wind anomalies can either increase or decrease a radiatively active species and thus QRS and QRL. Finally, as the atmosphere cools or warms, total longwave cooling rates will respectively decrease or increase (a negative feedback on temperature change).

**Fig. 3:**
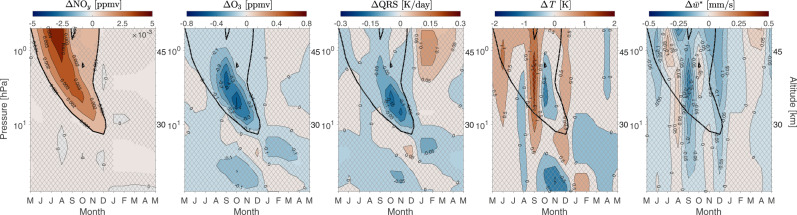
Stratospheric response to interannual variations in energetic particle precipitation (EPP). Nitrogen family, NO_*y*_ (NO_*x*_, HNO_3_, N_2_O_5_, ClONO_2_, HNO_4_), ozone (O_3_), short-wave heating rate (QRS), temperature (*T*) and vertical residual wind (${\overline{w}}{}^{*}$) for High EPP-Low EPP years in the SH polar region (60–90°S). Hatching indicates areas not statistically significant at 95% level based on the *t* test (*p* > 0.05). NO_*y*_ differences of 0.001 ppmv are marked with bold black contour. Pressure levels on the y-axis are 0.5–80 hPa, with approximate altitude in km given on the right-hand side. Contour intervals from left to right are 0.001 ppmv, 0.1 ppmv, 0.05 K day^−1^, 0.5 K, and 0.05 mm s^−1^. Contour color scales for each panel are given on the top of the panel.

We next explore the dependence of the ozone response to interannual variations in EEP. Fig. 3 shows the monthly mean differences between years with high and low EPP forcing (see “Methods”) for NO_*y*_, ozone, short-wave heating rate, temperature and the residual vertical wind. The tongue-like structure of descending NO_*y*_ during high EPP years, as seen in the first panel of Fig. 3 and overlaid in the remaining panels, coincides with ozone loss and springtime reduction in short-wave heating. The NO_*y*_ downward transport starts during winter (JJA), with enhancements of about 0.001 ppmv reaching altitudes below 30 km by October. The corresponding ozone loss of about 0.1–0.5 ppmv starts during the winter, lasting into early spring between 28 and 45 km. Ozone absorbs incoming solar radiation, which heats the atmosphere. Thus, the ozone loss results in the reduction of short-wave heating, at a rate of about 0.05–0.15 K day^−1^. However, any direct in situ temperature effect caused by ozone loss, i.e., stratospheric cooling, is mixed with impacts from other factors, namely anomalous vertical descent (negative anomalies in ${\overline{w}}{}^{*}$) which increase NO_*y*_, and drive dynamical heating that affects the total temperature response (Fig. 2, Pathway b).

**Fig. 4:**
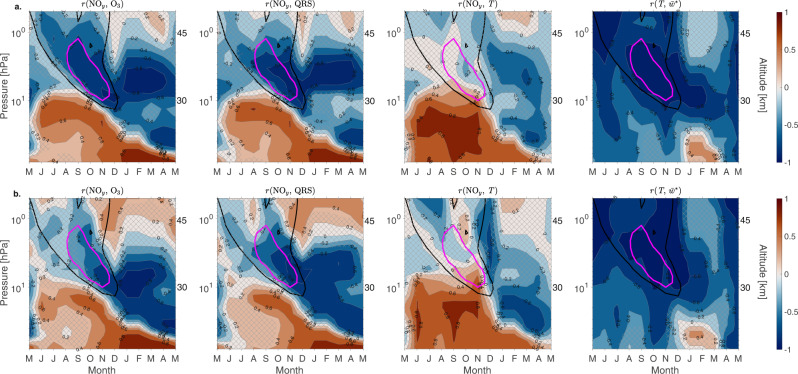
Connection between chemistry and dynamics. Correlation of monthly mean polar cap nitrogen family, NO_*y*_ (NO_*x*_, HNO_3_, N_2_O_5_, ClONO_2_, HNO_4_) and ozone (O_3_), shortwave heating rate (QRS), temperature (*T*) anomalies for (**a.**). High energetic particle precipitation (EPP) years, and (**b.**) Low EPP years. Last panels shows the correlation between temperature and ${\overline{w}}{}^{*}$ anomalies. Hatching indicates areas not statistically significant at 95% level (*p* > 0.05). NO_*y*_ differences of 0.001 ppmv from Fig. 3 are marked with bold black contour. Ozone differences of −0.2 ppmv from Fig. 3 are marked with magenta contour. Pressure levels on the *y*-axis are 0.5–80 hPa, with approximate altitude in km given on the right-hand side. Contour interval is 0.2. Contour color scales (same for all panels) are given on the right-hand side.

To better understand the connections between NO_*y*_, ozone, temperature and dynamics we examine correlations between NO_*y*_ and ozone, QRS, and *T* for High and Low-EPP years (Fig. 4a, b). For High-EPP years (Fig. 4a), the tongue-like structure of descending excess NO_*y*_ is clearly present in the negative correlation between NO_*y*_ and ozone from mid-winter into the spring period. The strongest correlation then persists at about 30 km until summer. Between August and December the correlation of NO_*y*_ and QRS in the upper stratosphere is also strongly negative; a result of the NO_*y*_-driven ozone loss and resulting reduction in shortwave heating. It would be reasonable to expect that the reduction in shortwave heating would result in decrease of temperature. However, we see no strong negative correlation between NO_*y*_ and temperature anomalies. Instead, *r*(NO_*y*_, *T*) is weak and largely statistically insignificant. Our simulations do show a negative correlation between NO_*y*_ and temperature between 30 and 40 km during the following summer (DJF). However, this does not correspond to the NO_*y*_-induced ozone loss and QRS reduction in this region (Fig. 3). The spatio-temporal patterns for Low-EPP years (Fig. 4b) are very similar to those presented in Fig. 4a. However, the correlations between NO_*y*_ and ozone, and NO_*y*_ and QRS, decrease significantly between August and November at altitudes above 30 km (marked with magenta contour) indicating the region of the EPP-NO_*y*_ driven chemical effect on ozone.

The large negative correlation between ${\overline{w}}{}^{*}$ and temperature anomalies between 28 and 45 km is similar for High and Low-EPP years (last column in Fig. 4a, b) and shows the strong relationship between vertical descent (ascent) and stratospheric heating (cooling) throughout the year. Thus the effect from vertical transport could potentially mask some, or all, of the radiative impact on temperature caused by ozone loss. These results indicate that the chemical effect of descending EPP-NO_*y*_ on ozone and the short-wave heating rate either have no direct significant impact on stratospheric temperatures in late winter/spring, or the influence is hidden in the overall dynamical variability. Below 10 hPa, the signals indicate a positive correlation between NO_*y*_ and ozone, and NO_*y*_ and QRS. This is consistent with previous studies and is likely related to NO_*y*_ interfering with chlorine-driven ozone depletion during winter/early spring seasons^27,28^.

### Time-slice sensitivity experiment: the role of medium energy electrons (MEE)

It has recently become evident that the descending NO_*y*_ reaching the stratosphere is underestimated in simulations. The two most probable reasons for that are (1) shortcomings in EPP forcing data sets^29–31^ and (2) mesospheric ion chemistry not being adequately represented in most models^32,33^. Both reasons contribute to the underestimation of EPP impact. Compared to the standard parameterization, inclusion of ion chemistry approximately doubles the amount of EPP-NO_*x*_ in the upper mesosphere during specific events. But even then NO_*x*_ levels are still largely underestimated when compared to the observations^34^. On the other hand, the currently recommended MEE forcing data set (see “Methods”) provides a lower range of precipitating fluxes into the atmosphere^29^. Together, more comprehensive EPP fluxes and mesospheric ion chemistry, would most likely solve the underestimation problem.

As an indication of the issue, when compared to the springtime stratospheric EPP-NO_*y*_ derived from satellite observations^22^, the monthly mean anomalies from our transient simulation are up to an order of magnitude smaller (Fig. 3). At 45 km/30 km, the average amount of observed EPP-NO_*y*_ in 2002–2012 is between 0.02 and 0.06 ppmv/0.006 and 0.02 ppmv. In our simulations at the same altitudes, NO_*y*_ anomalies for high-low EPP years are on average 0.002 and 0.004 ppmv and 0.001 ppmv, respectively.

**Fig. 5:**
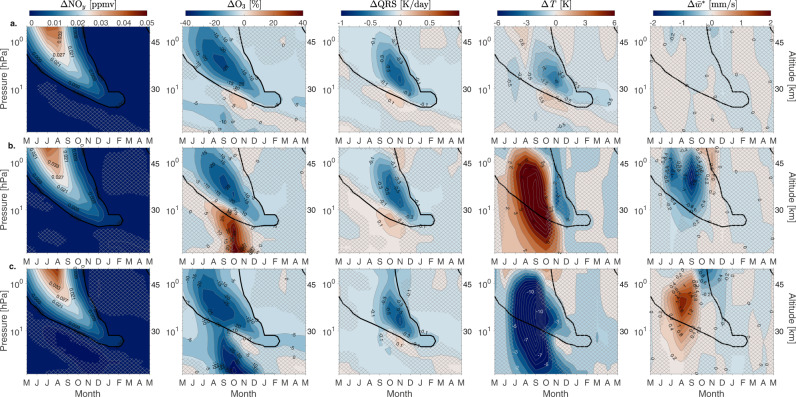
Time-slice sensitivity experiment. Monthly mean differences of SH polar (60–90°S) nitrogen family, NO_*y*_ (NO_*x*_, HNO_3_, N_2_O_5_, ClONO_2_, HNO_4_), ozone (O_3_), shortwave heating rate (QRS), temperature (*T*) and residual vertical wind (${\overline{w}}{}^{*}$) (from left to right) for the idealized experiments labeled FW^max^ and FW^min^: (**a.**) FW^max^-FW^min^, (**b.**) FW^max^-FW^min^ for years with strong negative residual vertical wind differences, ${\overline{w}}{}^{*}$ (downwelling), and **(c.)** FW^max^-FW^min^ for years with strong positive residual vertical wind differences, ${\overline{w}}{}^{*}$ (upwelling). Hatching indicates areas not statistically significant at 95% level based on the *t* test. The number of years used for computing the composite differences: (**a**) 50/50 years, (**b**, **c**) 10/10 years. NO_*y*_ differences of 0.003 ppmv are marked with bold black contour. Pressure levels on the *y*-axis are 0.5–80 hPa, with approximate altitude in km given on the right-hand side. Contour intervals for the columns from left to right are 0.003 ppmv, 5%, 0.1 K day^−1^, 0.5 K (**a**) and 1 K (**b**, **c**), and 0.2 mm s^−1^. Contour color scales for each column of panels are given at the top.

Therefore, we can argue that the lack of an EPP-NO_*y*_ driven temperature response in the stratosphere in our simulations is a consequence of driving the model with EPP that is too low and produces too little NO_*y*_. To test the impact of a more realistic EPP-NO_*y*_ amount we completed two additional 50-year model simulations with fixed high and low MEE fluxes under perpetual year 2000 constituent lower boundary conditions (see “Methods” and Table 1). The results are shown in Fig. 5. Comparing the monthly mean differences between the simulations with high and low EPP forcing to observations^22^, the amount of EPP-NO_*y*_ in the stratosphere is now close to the observed levels excluding the years of anomalously high and low values. Thus this experiment allows us to assess the EPP dynamical impact in more realistic NO_*y*_ conditions than possible using the transient simulations.

**Table 1:** Solar irradiance and energetic particle precipitation (EPP) forcing terms for the idealized experiments labeled FW^max^ and FW^min^

Set	Description	*Ap*	*Kp*	*F*10.7/*F*10.7*a*	MEE	SPE, GCR
FW^max^	high solar irradiance, high EPP	27	4	210/210	10 × 2003 mean	–
FW^min^	low solar irradiance, low EPP	1	0.1	40/40	–	–

*Ap and Kp* are indices of geomagnetic activity, MEE is medium energy electrons, SPE is solar proton event, GCR is galactic cosmic rays. *F*10.7/*F*10.7a is solar radio flux reported in solar flux units, sfu = 10^−22^ Wm^−2^ Hz^−1^.

In Fig. 5a, the tongue-like structure of descending EPP-NO_*y*_ is again seen, and the enhanced NO_*y*_ of about 0.003 ppmv reaches altitudes below 30 km by August. This corresponds to statistically significant ozone loss, up to 25%, at altitudes between 28 and 45 km during late winter/spring, similar to what has been seen in observations^5^. Ozone loss is followed by a significant reduction in short-wave heating rates of about 0.6 K day^−1^. The expected cooling of the stratosphere due to ozone heating reduction is now significant and ranges from 1.5 to 3 K at altitudes between 28 and 35 km during the spring. In these averages, the dynamical variability (${\overline{w}}{}^{*}$) is negligible and does not mask the radiative effect from EPP-NO_*y*_ and chemistry. These results indicate that a realistic representation of mesospheric EPP-NO_*y*_ leads to clear response in stratospheric temperature.

Assessing the impact of EPP on stratospheric temperature is challenging and demands long simulation time series in order to determine statistically robust signals from background dynamical variability. As an example, Fig. 4b, c shows the differences between time-slice simulations of high and low EPP forcing when selecting years in such a way that the two data sets have (1) strong negative differences and (2) strong positive differences in residual vertical wind, ${\overline{w}}{}^{*}$ (see “Methods”). In other words, we are now comparing simulations with very different background dynamical conditions. The amount of EPP-NO_*y*_ in both cases is very similar to the average amount in Fig. 5a, again causing up to 25% of ozone loss and up to 0.5 K day^−1^ reduction in short wave heating. The ozone and QRS responses being similar above 10 hPa in all cases is further evidence of a chemical effect from NO_*y*_ increase. However, anomalous variability in temperature (increase or decrease) associated with the vertical motion (descent or ascent) makes the EPP signal difficult to detect (Fig. 5b, c).

The positive (negative) temperature effect in Fig. 5b (5c) is a manifestation of dynamical heating (cooling). To demonstrate this we calculated the rates of dynamical heating and cooling associated with vertical motion along with the long-wave cooling rates, QRL (Supplementary Fig. 1). Heating rates associated with vertical motion are calculated using Eq. (2) (see “Methods”). The results show that anomalous vertical descent acts to dynamically heat the stratosphere, which is then balanced by radiative cooling (Supplementary Fig. 1a, Pathway ab). The temperature increase associated with dynamical heating peaks in September-October (Fig. 5b). Enhanced upwelling acts the opposite way (Supplementary Fig. 1b, Pathway cd). Dynamical term in both cases is dominant while EPP-induced changes in QRS (Fig. 5b, c) are much smaller.

The large dynamical differences mask the smaller EPP effect which would be detectable otherwise. Averaging over all 50 years of the time-slice scenario averages out the noise arising from dynamical variability, now revealing the EPP signal (Fig. 5a). Similar challenges in EPP signal detection were present in the transient simulations (Fig. 3). While increasing the magnitude of EPP-NO_*y*_ is important for statistically significant temperature response, our results suggest that a composite difference between data sets of around 50 years or more is necessary to average out the dynamical variability. The composite differences in our transient simulation that consisted of 36 years for high EPP and 33 years for the low EPP, as well as composite differences in time-slice experiment with different background dynamical conditions (10 years each) were not sufficient.

Our analysis also suggests that the differences of lower stratospheric ozone in late winter (up to ± 40% below 20 hPa) is not EPP-related but most likely due to differences in background dynamics. For example, the cooler temperatures at these altitudes in Fig. 5c likely result in enhanced polar stratospheric cloud (PSC) formation and heterogeneous chlorine activation that leads to polar ozone depletion. Since we do not see clear NO_*y*_ differences at these times and altitudes between high and low EPP cases we can rule out that this is an EPP-ozone response.

## Discussion

Resolving the existing inconsistency between the expected effect on springtime ozone of EPP and the subsequent stratospheric temperature change^24^ is a crucial step towards understanding the EPP impacts on the atmospheric dynamics.

When our simulated NO_*y*_ agrees with observed amounts and dynamical heating effects have been accounted for, we clearly see a stratospheric cooling related to EPP ozone loss and our results are in line with the other modeling and observational studies^10,12,17^. This highlights the shortcomings of the current MEE/EPP forcing data and the urgent need to improve it by introducing new and better satellite-based observations of EPP fluxes.

When applying MEE/EPP according to our current understanding, it seems clear that covariance between EPP-NO_*y*_ and late winter/springtime temperature is partly driven by radiative effects via ozone destruction, but mainly responds to the changes in the mean vertical wind over the polar area. Therefore, assessing EPP-NO_*y*_ impacts on temperature in the stratosphere requires not only more accurate MEE/EPP forcing datasets, but also long simulations for a statistically robust separation of any EPP signals from the background dynamical variability.

The descent of EPP-NO_*y*_ driving stratospheric ozone loss during late winter/spring is one of the several mechanisms that could initiate a dynamical response to EPP in the troposphere. Two other mechanisms include: the EPP-NO_*y*_ impact on stratospheric ozone during mid-winter (less long-wave cooling → stratospheric warming) and EPP-HO_*x*_ impact on mesospheric ozone with resulting influence to dynamics during the winter. All mechanisms together are linked to polar stratospheric dynamics and could play a role in modulating regional-scale ground-level climate. Improving our current knowledge of the MEE/EPP forcing data and its magnitude is a necessary step to understand linking mechanisms and their impacts on the regional climate variability.

## Methods

### Model description

WACCM is a 3D chemistry-climate model that extends from the surface to 5.9 × 10^−6^ hPa (~140 km) with horizontal resolution 1.9° latitude by 2.5° longitude. Here we use version 4 of the model^25,26^ with the standard photochemistry setup (52 neutral and five ionic species) and a lookup table parameterization for HO_*x*_ and NO_*x*_ production from EPP^35,36^.

### Transient simulations

In the first part of our analysis we investigate the dynamical effects of the indirect EPP-NO_*y*_ using model setup ("compset" B55TRWCN) that includes active ocean and sea-ice components. An ensemble of three WACCM transient simulations was carried out with all observed forcing from 1957 to 2005. The three ensemble members (49 years each) result in 147 years in total for our analysis. The initial conditions for 1957 for all model components were taken from a single historical simulation (1850–2005). The setup is identical to the Coupled Model Intercomparison Project Phase Five (CMIP5)^26^ (including ionization from SPE and auroral electrons), but with added solar forcing via MEE. The MEE forcing used represents electron precipitation in the energy range of 30–1000 keV, and is recommended for the CMIP6^29,37^. We utilize MEE model driven by the observed geomagnetic *Ap* index^29,38^. In the model, *Ap* defines the level of magnetospheric disturbance and the location of the plasmapause, both of which are needed to calculate precipitating electron fluxes. The daily, zonal mean fluxes of precipitating electrons from the model were used to calculate atmospheric MEE-driven ionization rates included in WACCM. GCR ionization is not included as its effect above 10 hPa is negligible. To minimize underlying ozone anomalies below 30 km altitude related to the development of the ozone hole, the anomalies in this part of the analysis were calculated by (1) dividing the years into two periods: pre-ozone hole years (1957–1981) and ozone hole years (1982–2005), and (2) subtracting the corresponding climatological monthly mean values for 1957–1981 and 1982–2005, respectively. Following the approach of Seppälä et al. (2009, 2014)^9,17^ and Andersson et al. (2018)^38^, we have chosen to use the geomagnetic *Ap* index as a proxy for the level of EPP in order to account for a wide range of particle energies in our analysis. Based on that, we have selected high EPP years: 1957–1960, 1974, 1982–1984, 1989, 1991, 1994, 2003 and Low EPP years: 1964–1966, 1969–1971,1980, 1987, 1996–1998. Note, however, that the amount of EPP-produced NO_*y*_ in the stratosphere also depends on meteorological conditions^23^. Correlation analysis was done using Pearson correlation coefficients calculated over the period of for High-EPP years (36 in total) and Low-EPP years (33 in total) for each month and pressure level using polar cap averages. All correlations are calculated using anomalies.

Residual vertical wind, ${\overline{w}}{}^{*}$ is calculated for every time step using Eq. 3.5.1b in Andrews et al. (1987)^39^, i.e.,

1 $${\overline{w}}{}^{*}\equiv \overline{w}+{(a\cos \phi )}^{-1}{(\cos \phi \,\overline{{v}^{{\prime} }{\theta }^{{\prime} }}/\overline{{\theta }_{z}})}_{\phi },$$

where *w* is the vertical wind, *a* is Earth’s mean radius, *ϕ* is latitude, and $\overline{{v}^{{\prime} }{\theta }^{{\prime} }}$ (where *v* is the meridional wind and *θ* is potential temperature) is the eddy heat flux term. Bar denotes zonal mean, and ${}^{{\prime} }$ indicates deviation from the zonal mean.

### Time-slice sensitivity experiment

In the second part, we focus on the role of EPP-NO_*y*_ induced effects on atmospheric dynamics and the importance of an adequate representation of MEE ionization. The model is used with prescribed ocean and sea-ice components and present day atmospheric conditions (compset FW). This is an idealized time-slice experiment to test our hypothesis on the impact of different levels of EPP-NO_*y*_ on chemical-dynamical coupling. To bound the source of NO_*x*_ from EPP, two 50-year experiments were conducted: (1) high MEE (10 × mean of year 2003) and solar irradiance maximum conditions (FW^max^), and (2) low MEE and solar irradiance minimum conditions (FW^min^). The *Ap* and *Kp* indices as well as the 10.7 cm solar radio flux (F10.7) are given in Table 1. The *Kp* is a planetary index of geomagnetic activity. The mean MEE fluxes are based on the CMIP6 solar forcing^37^. For the FW^max^ case, we increased the 10.7 cm flux as high EPP years often occur during high solar irradiance years^17^. As these are hypothesis testing sensitivity experiments, the MEE and irradiance are kept constant throughout each simulation. SPEs or GCR are not included in the sensitivity studies. Apart from the solar input, all other parameters in these sensitivity simulations are identical. We increase the EPP forcing to match the observed amount of NO_*y*_ in our simulations, to allow for a more accurate magnitude of the EPP impact on ozone and dynamics. The criteria for the high negative and high positive ${\overline{w}}{}^{*}$ differences are (1) the ${\overline{w}}{}^{*}$ differences between FW^max^ and FW^min^ are negative or positive (at least 0.7 mm s^−1^) and (2) the negative or positive differences in ${\overline{w}}{}^{*}$ are not changing sign abruptly.

Heating rates associated with vertical motion are calculated as

2 $${\overline{w}}{}^{*}(H{N}^{2}/R+\partial \overline{T}/\partial z),$$

where *H* is the mean scale height, *N* is the Brunt-Väisälä frequency, *R* is the gas constant, with the *HN*^2^/*R* term representing global mean static stability. $\overline{T}$ here is the zonally averaged deviation from global mean temperature^40^.

## Supplementary information

Supplementary Information

## Data Availability

The processed model data used in this study are available on Zenodo (10.5281/zenodo.3581408)^41^.

## Code availability

CESM source code is distributed through a public subversion code repository (http://www.cesm.ucar.edu/models/cesm1.0/).
