# Supplementary material for: Ozone impact from solar energetic particles cools the polar stratosphere
Source: Nat Commun. 2022 Nov 12;13:6883. doi: 10.1038/s41467-022-34666-y (PMC9653381; doi:10.1038/s41467-022-34666-y)
Supplement: Supplementary file 1 — Supplementary Information [file 41467_2022_34666_MOESM1_ESM.pdf]

Ozone impact from solar energetic particles cools the polar stratosphere: supplementary information

Monika E. Szeląg[1,*], Daniel R. Marsh[2,3], Pekka T. Verronen[1,4], Annika Seppälä[5], and Niilo Kalakoski[1]

[1]Space and Earth Observation Centre, Finnish Meteorological Institute, Helsinki, Finland.
[2]Climate and Global Dynamics Laboratory, National Center for Atmospheric Research, Boulder, CO, USA.
[3]Faculty of Engineering and Physical Sciences, University of Leeds, Leeds, UK.
[4]Sodankylä Geophysical Observatory, University of Oulu, Sodankylä, Finland.
[5]Department of Physics, University of Otago, Dunedin, New Zealand.
[*]monika.szelag@fmi.fi

Content

1. Supplemantary Figure 1

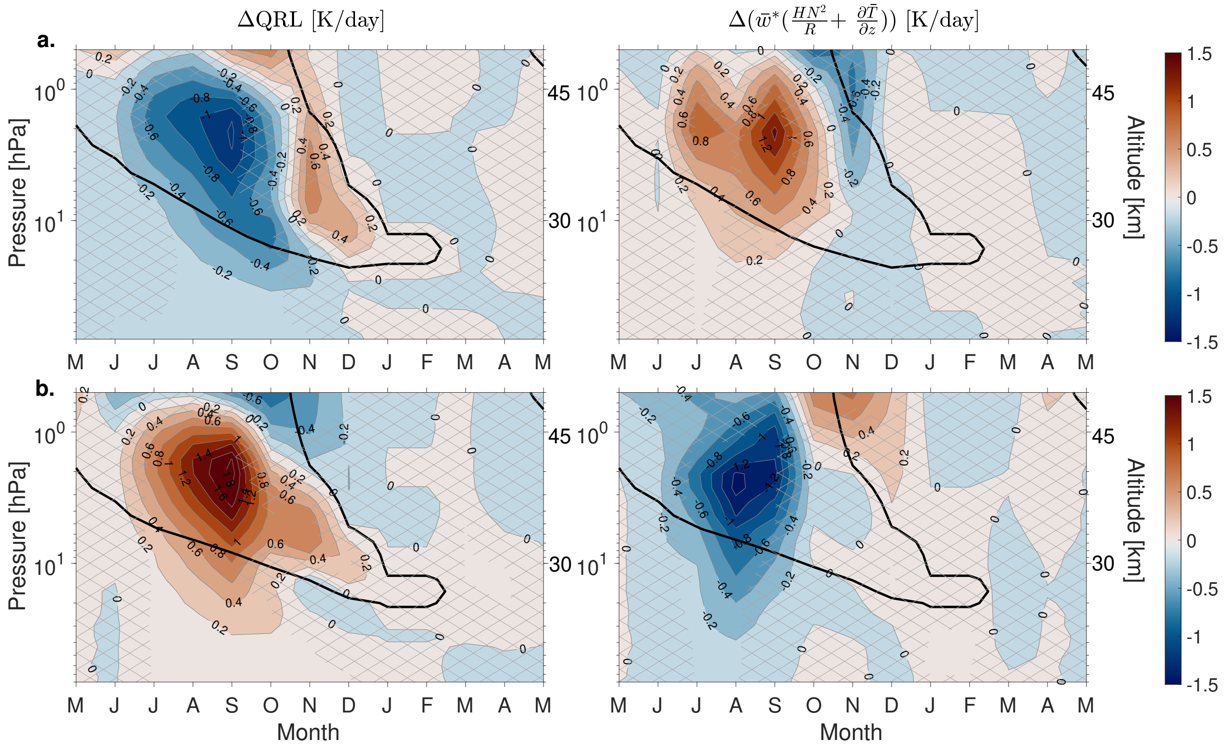

Supplementary Figure 1. Background dynamical variability. Monthly mean differences of SH polar (60–90°S) long wave heating rates (QRL) and dynamical heating/cooling rates ($\overline{w}^*$ $(\frac{HN^2}{R} + \frac{\partial \overline{T}}{\partial z})$) for the idealised experiments labelled FW^{max} and FW^{min}: **(a.)** time-slice experiment, FW^{max}-FW^{min} for years with strong negative residual vertical wind differences, $\overline{w}^*$ (downwelling), and **(b.)** time-slice experiment, FW^{max}-FW^{min} for years with strong positive residual vertical wind differences, $\overline{w}^*$ (upwelling). HN^2/R represents the global mean static stability and $\overline{T}$ is the zonally averaged deviation from global mean temperature. Hatching indicates areas not statistically significant at 95% level based on the t-test. The number of years used for computing the composite differences is 10/10 years. NO_y differences of 0.003 ppmv from Fig. 5 (main manuscript) are marked with bold black contour. Pressure levels on the y-axis are 0.5–80 hPa, with approximate altitude in km given on the right hand side. Contour interval is 0.2. Contour colour scales (same for all panels) are given on the right hand side.
